# Do Diabetes Mellitus Differences Exist within Generations? Three Generations of Moluccans in The Netherlands

**DOI:** 10.3390/ijerph18020493

**Published:** 2021-01-09

**Authors:** Adee Bodewes, Charles Agyemang, Anton E. Kunst

**Affiliations:** Department of Public and Occupational Health, Amsterdam UMC, University of Amsterdam, 1105 AZ Amsterdam, The Netherlands; c.o.agyemang@amsterdamumc.nl (C.A.); a.e.kunst@amsterdamumc.nl (A.E.K.)

**Keywords:** diabetes, migrants, three generations, Moluccans, The Netherlands

## Abstract

*Background:* Diabetes mellitus (DM) is known to be more prevalent among migrants compared to their host populations. It is unclear whether DM prevalence differs between generations among migrants. We investigated the differences in DM prevalence among three generations of Moluccans, who have been living for over 65 years in the Netherlands, compared to the Dutch population. *Methods*: In this cross-sectional study, data of a healthcare insurance database on hospital and medication use (Achmea Health Database) were used. The dataset contained 5394 Moluccans and 52,880 Dutch persons of all ages. DM differences were assessed by means of logistic regression, adjusting for age, sex, urbanization, and area socio-economic status. *Results*: The prevalence of DM was higher in all generations of Moluccans compared to the Dutch. The adjusted odds ratios (AORs) for DM were significantly higher in total group of Moluccans compared to the Dutch (AOR 1.60, 95% CI 1.42–1.80) and across the first and second generation of Moluccans compared to the Dutch (first generation (1.73, 1.47–2.04) and second generation (1.44, 1.19–1.75). Higher AOR were found for first generation men (1.55, 1.22–1.97) and first (1.90, 1.52–2.37) and second (1.63, 1.24–2.13) generation Moluccan women compared to the Dutch. AOR for the third generation Moluccans was increased to a similar extent (1.51, 0.97–2.34), although not statistical significant. *Conclusions*: Our findings show higher odds of DM across generations of Moluccans compared to the Dutch. DM prevention strategies for minorities should be targeted at all migrant generations in host countries.

## 1. Introduction

Migrants have a higher prevalence and burden of diabetes mellitus (hereafter DM) compared to their host populations [1,2,3,4,5,6,7]. DM is projected by the WHO to be one of the leading causes of death by the year 2030 [8]. In the Netherlands, increased DM prevalence rates are found among the Turkish, Moroccan [9,10,11], Surinamese [10,12], and Ghanaian migrants [13,14] compared to the host Dutch population. These migrant groups mainly have DM type 2 and have on average two to five times higher odds of DM compared to the Dutch population, and the DM age of onset for Turkish and Moroccans is one or two decades earlier. This is partly explained by the high prevalence of overweight and obesity and a low socioeconomic status among these migrant groups compared to the Dutch population. It is suggested that these migrant groups make less effective use of DM care, which leads to more complications and an increase of healthcare costs [6,9,11,13,14,15,16].

Several studies have hypothesized that the first generation have the highest prevalence of DM compared to their offspring. Several first generation migrants from low- and middle-income countries have been raised in a food scarce environment, suggesting that their bodies are programmed to cope with limited food supply [3,4]. Later in life, DM risk increases, as migrants may be at high risk of rapid weight gain in the food abundant environment of high-income countries. The next generation of these migrants, who were born in the host country, were not exposed to such environmental change, and are therefore expected to have lower or similar levels of DM risk compared to the host population [4]. Ho et al. found evidence for higher DM mortality rates among first generation Indonesians and likely lower DM mortality among the second generation Indonesians compared to the host Dutch population [17]. Yet, knowledge is lacking regarding DM prevalence rates for both DM types in the younger generations. In addition, evidence for DM implications among younger generations is lacking. There is an urgent need for more evidence on DM among different generations to get more insight into the burden of DM among migrant groups.

In the Netherlands, there are multiple generations of Moluccan migrants. The Moluccans arrived in the Netherlands in 1951. They were soldiers serving the Royal Dutch East-Indies Army during world war II [18,19]. The Moluccans were brought ”temporarily” to the Netherlands in the 1950s and were expected to return to country of origin. A decade after arrival, they were placed in specific Moluccan residential areas, so-called ”Moluccan districts”. Overall, the Moluccans are lower educated and have lower-ranking occupations compared to the Dutch [18,19].

Therefore, the objective of this study was to assess the differences in DM prevalence (including both type 1 and type 2 DM) between three generations of Moluccans compared to the Dutch population. The first generation has been living for over 60 years in the Netherlands, and no large remigration has occurred, so comparisons can be made with the younger generations.

Our hypothesis was that the prevalence of DM would be higher in first generation than second and third generation Moluccan migrants because of their exposure to a poor socio-economic environment during early life, military service during the World War II, and exposure to an obesogenic environment post migration in adulthood. Furthermore, the expectation of returning to their country of origin was not fulfilled by the Dutch government. Consequently, especially the first generation Moluccans might have endured (long) periods of stress and/or depression expressions associated with integration [20].

## 2. Materials and Methods

### 2.1. Study Population

In this cross-sectional study, we used registered health insurance declarations, within the period of 1 January 2009, to 31 December 2010, from a healthcare insurance database on hospital and medication use (Achmea Health Insurance database) of Moluccans and the Dutch population [21]. Achmea is the largest health insurance company of the Netherlands, covering approximately 40% of the total population. Every Dutch citizen is required by law to have health insurance [19].

A list of more than 1700 Moluccan family names was used to select Moluccans. This list was made when the Moluccans arrived in the Netherlands in 1951. The first four letters of the Moluccan family name were used for selection, and variations of the family names were taken into account. The first four characters of the surnames were used for selection because of privacy reasons. A check among the surnames showed that Moluccan surnames could be distinguished from the Dutch surnames based upon these four characters. A number of 5532 Moluccans could be identified (number of men: 2658; women: 2736; a mean age of 38.7 years). A random Dutch native’s sample of ten times the number of Moluccans, 55,320 persons, was extracted, matched on sex and age (number of men: 26,003; women: 26,877; a mean age of 38.7 years).

No ethics approval was needed according to the medical ethical commission of the Academic Medical Centre, Amsterdam (AMC) (reference number W13-031# 13.17.0045), as this research is not subjected to the Medical Research Involving Human Subjects Act (WMO). All patient records were anonymized and de-identified prior to analysis.

### 2.2. Measurements

For each case we had the following variables: ethnicity, age, sex, area socio-economic status (SES), urbanization, number of medical specialist and general practitioner (GP) consultations, and days insured. Additionally, for each case the declared medical procedure(s) and medication treatment were available by specific Diagnosis Treatment Combination code (DTC code) and Anatomical Therapeutic Chemical classification code (ATC code), respectively.

Area-SES was based on a list of SES scores per postal code area in the Netherlands, as stated by the Netherlands Institute for Social Research (Sociaal Cultureel Planbureau, SCP). This score was composed based on the mean education, income, and employment level.

Urbanization was estimated based on four number postal codes. The classification of the Statistics Netherlands (Centraal Bureau voor Statistiek, CBS) was used, which is a five-point scale from “non urbanized” to “highly urbanized” areas.

### 2.3. Data Handling and Analysis

The primary outcome was the clinical diagnosed prevalence of overall DM including type 1 and 2. We excluded all persons insured less than one whole year (365 days) from all analyses, to make the study groups comparable. A combination of the Diagnosis Treatment Combination (DTC) and Anatomical Therapeutic Chemical Classification System (ATC) code was used to select all persons with DM. This selection method provided the most accurate estimate of the number of persons with DM. However, we were not able to distinguish between type 1 and type 2 DM.

We standardized all prevalence rates for age, based on the age distribution of the Moluccan group. The Moluccans and the Dutch were divided into three birth generation groups. The birth generation groups were based upon the time at arrival (1951) and the mean age at first birth (1976: 19.4 years) in Indonesia [22]. The first generation migrants were born before 1950, the second generation migrants were born between 1951 and 1970, and the third generation were born after 1970. Logistic regression was used to assess the differences in the odds of having DM between Moluccans and the Dutch population. Two models were used to examine the data. Model 1 adjusted for age and sex and model 2 additionally adjusted for area-SES and urbanization. A *p*-value ≤ 0.05 was considered to be statistically significant. The Statistical Package for the Social Sciences (SPSS) version 24.0 (IBM, New York, NY, United States) was used for all analyses.

## 3. Results

Table 1 shows the distribution of characteristics for the Moluccan and the Dutch population. Moluccans lived more often in lower SES areas than the Dutch (SES percentile <5: 65.1%; Dutch 49.7%). More Moluccans lived in highly urbanized areas (38.5%, Dutch 30.4%), whereas the Dutch population lived more often in rural areas (5.6%, Moluccan 1.1%). More third generation Moluccans (41.6%) lived in highly urbanized areas compared to the first (35.7%) and second (35.2%) generation Moluccans.

Table 2 shows the DM prevalence across generations among the Moluccans and the Dutch. High DM prevalence rate was found among Moluccans (7.0%) compared to the Dutch (4.5%). Additionally, for each generation group, the Moluccans had higher DM prevalence compared to the Dutch.

Table 3 shows the DM odds across generation and by sex among the Moluccans and the Dutch. Moluccans had higher odds of DM after adjusted for sex and age (OR: 1.67, 1.48–1.88), area-SES, and urbanization (OR: 1.60, 1.42–1.80). Significant higher odds of DM were observed in the first and second generation groups (OR: first generation 1.84, 1.56–2.16; second generation 1.48, 1.22–1.80) compared with Dutch. The differences persisted after further adjustment for area-SES and urbanization. Odds for the third generation Moluccans were increased to a similar extent as for the second generation (1.51, 0.97–2.34). However, as 95% confidence intervals were wider, due to smaller number of cases of DM at young ages, the difference was not statistically significant.

Moluccan men (1.43, 1.20–1.71) and particularly women (1.77, 1.50–2.08) had higher odds of DM compared to the Dutch after adjusted for age, area-SES, and urbanization. Significant higher DM odds were found for the first and second generation men (first generation: 1.61, 1.27–2.05; second generation: 1.31, 1.00–1.74) and women (first generation: 2.07, 1.66–2.57; second generation: 1.68, 1.28–2.19) compared to the Dutch. Third generation Moluccan men and women both had higher odds, though not statistically significant.

**Figure 1:**
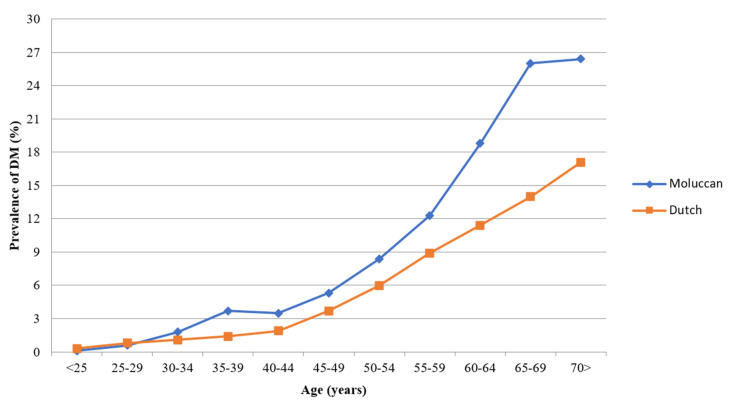
DM age of onset for Moluccans and Dutch population (%).

Figure 1 shows the DM age of onset for Moluccans and the Dutch population. The DM age of onset seems to be five years earlier for Moluccans aged 45 to 55 years compared to the Dutch.

## 4. Discussion

The first generation Moluccans have significantly higher DM odds compared to the Dutch. Additionally, across the younger generations, Moluccans have higher odds of DM compared to the Dutch. Both Moluccan men and women show higher DM odds compared to the Dutch and across generations.

We expected a higher prevalence of DM among the first generation than second and third generation Moluccans by reasoning that the first generation was exposed to poor socio-economic environment during early life, served for years during the World War II, and was exposed to obesogenic environment post migration in adulthood. Our results indeed show a relatively large difference for the first generation. However, the second and third generations of Moluccans also have high odds of DM. This implies that the high DM prevalence found across generations cannot solely be explained by early life exposures, but rather other factors such as lifestyle and genetics, known risk factors for DM may also play a role [4,7,13,15,23,24,25].

The preservation of the Moluccan lifestyle in terms of traditional food may contribute to the high DM prevalence rates among generations of Moluccans. The Moluccan culture is well preserved within the community. The Moluccan traditional cuisine contains an excess of fat, sugar, and salt. As a result, Moluccans tend to have more overweight (men: 49.0%; women: 35.0%) than the Dutch population (men: 46.4%; women: 30.5%), although physical activity levels are similar [26].

Genetic predispositions may also contribute to the observed high prevalence of DM across generations of Moluccans [27,28]. Chambers et al. have identified genetic variations among South Asians such as a gene cluster that is linked to core metabolic traits of DM [29]. Such gene clusters may well be found in the Moluccan genome. As no research data are available on this topic for Moluccans, further research is explicitly needed. The Moluccans originate from Southeast Asia, by which the possible explanations found among South Asians may also be applicable for the Moluccans.

DM is problematic across generations of Moluccans, men and women, in the Netherlands. Our results showed higher DM odds even among the younger generation Moluccans, where the DM age of onset seems to be about five years earlier compared to the Dutch.

### Strengths and Limitations

A major strength of this study is the use of claimed healthcare data, which gave us the unique opportunity to investigate registered persons with DM among different generations of Moluccans. Additionally, this is the first study to give an overview of DM among three generations of Moluccans. Up until now, no DM data were available among this group.

Several limitations should be taken into account when interpreting our findings. First, the selection method might have limited us to select all Moluccan people. Some Moluccan women married to Dutch men and their children might have been excluded from the selection, as the last names of these women and children, in most cases, would be Dutch. However, another selection method was not feasible, as Moluccans could not be identified in population registries. This could have resulted in underrepresentation of Moluccans who were more integrated or even assimilated into Dutch society.

Second, the Achmea Health Database only contains information on claimed healthcare costs. People were rated having DM when they had claimed specialized DM care. This could have resulted in an underestimation of DM, considering that not all people with DM seek specialized care. This may be the case for Moluccans, as they tend to seek less medical care compared to the Dutch, which may result in an underestimation of the found DM differences.

Third, we could not make a distinction between DM type 1 and type 2 in this study, as DBC codes for the different DM types were not available at the time of data collection. Such distinction would have provided essential information for measuring and understanding inequalities in both DM type 1 and type 2 prevalence rates. We expect that our findings mostly reflect patterns for DM type 2, as this type is the most common among other minority groups in the Netherlands [7,30].

Lastly, the healthcare registry did not include information on explanatory factors such as SES scores or obesity. In this study, we could use a proximate SES score based on the place of residence. Such an area-level SES score partly reflects the SES of individual persons, but may not fully capture individual-level variations in education or income. In the Netherlands, Moluccans in all generations have on average a lower educational level and a lower household income compared to the Dutch [31]. By using only an area-SES score, we may have underestimated inequalities in education and income, and their contribution to the observed inequalities in DM risk. Moreover, due to lack of information on specific risk factors such as obesity, we could not assess the extent to which these have contributed to the higher DM prevalence of Moluccans.

## 5. Conclusions

We found high DM prevalence rates across generations of Moluccans compared to the Dutch. To reduce DM risk among both younger and older Moluccans, we recommend the implementation of community-based lifestyle interventions targeted at all generations. Further research should identify the factors influencing DM risk and prognosis across generations of migrants in order to inform DM prevention and clinical management strategies for different generations.

## Figures and Tables

**Table 1:** Age-standardized distribution of characteristics among Moluccans and the Dutch population in total and by generation (%).

	Total		Generation		
	Dutch	Moluccans	1st Generation	2nd Generation	3rd Generation
	*n* = 52,880	*n* = 5394	*n* = 854	*n* = 1737	*n* = 2803
Sex					
Men	49.2	49.3	44.8	49.0	51.0
Women	50.8	50.7	55.2	51.0	49.0
Age					
<25	30.2	30.1	-	-	61.2
25–39	20.7	21.2	-	-	38.8
40–60	31.3	31.9	-	100	-
>60	17.8	16.8	100	-	-
Area-SES (percentile)					
1 (lowest)	9.7	15.3	12.6	12.6	18.0
2	10.2	16.9	18.6	16.4	16.7
3	9.7	14.3	14.0	14.9	14.1
4	10.2	7.9	6.5	7.3	8.1
5	9.9	10.7	13.2	11.2	11.0
6	9.9	7.5	8.7	7.5	7.0
7	10.4	5.8	5.7	5.1	5.7
8	10.1	7.9	8.7	9.0	6.7
9	10.0	6.1	6.4	7.2	5.1
10 (highest)	10.0	7.5	5.7	8.7	7.6
Degree of urbanization					
1 (rural)	5.6	1.1	0.8	1.2	1.2
2	17.7	18.1	24.3	20.1	14.6
3	17.6	15.4	15.4	16.5	14.7
4	28.6	26.9	23.7	26.8	28.0
5 (highly urban)	30.4	38.5	35.7	35.2	41.6

**Table 2:** DM age-standardized prevalence rates between Moluccans and the Dutch population (ref.) in total and by generation.

	Total		1st Generation		2nd Generation		3rd Generation	
	*n*	% DM	*n*	% DM	*n*	% DM	*n*	% DM
Dutch	52,880	4.5	9782	14.4	16,620	5.4	26,478	0.6
Moluccans	5394	7.0	958	23.2	1714	7.6	2722	0.9

**Table 3:** DM odd ratios (OR) between Moluccans and the Dutch population (ref.) in total and by generation.

OR (95% CI)	Total	1st Generation	2nd Generation	3rd Generation
Dutch	1.00	1.00	1.00	1.00
Moluccan				
Adjusted for a, b	1.67 * (1.48–1.88)	1.84 * (1.56–2.16)	1.48 * (1.22–1.80)	1.48 (0.96–2.28)
Adjusted for a, b, c, d	1.60 * (1.42–1.80)	1.73 * (1.47–2.04)	1.44 * (1.19–1.75)	1.51 (0.97–2.34)
Men				
Adjusted for a	1.47 * (1.23–1.75)	1.61 * (1.27–2.05)	1.31 * (1.00–1.74)	1.41 (0.72–2.74)
Adjusted for a, c, d	1.43 * (1.20–1.71)	1.55 * (1.22–1.97)	1.28 (0.97–1.70)	1.46 (0.74–2.86)
Women				
Adjusted for a	1.87 * (1.59–2.20)	2.07 * (1.66–2.57)	1.68 * (1.28–2.19)	1.54 (0.87–2.72)
Adjusted for a, c, d	1.77 * (1.50–2.08)	1.90 * (1.52–2.37)	1.63 * (1.24–2.13)	1.55 (0.87–2.75)

* Significant differences: all *p*-values: < 0.05. Adjusted interaction term ethnicity * generation showed *p*-value = 0.150. Adjusted for confounders: a—Age; b—Sex (men reference group); c—Area-SES (per unit increase); d—Urbanization (per unit increase).

## Data Availability

The dataset supporting the conclusions of this article are available, on request, in the institution repository of the AMC in a secured internal environment.
